# Childhood Health Status and Adulthood Cardiovascular Disease Morbidity in Rural China: Are They Related?

**DOI:** 10.3390/ijerph13060565

**Published:** 2016-06-06

**Authors:** Qing Wang, Jay J. Shen

**Affiliations:** 1 School of Business, Dalian University of Technology, Panjin 124221, China; 2 Department of Health Care Administration and Policy, School of Community Health Sciences, University of Nevada at Las Vegas, Las Vegas, NV 89103, USA; jay.shen@unlv.edu

**Keywords:** childhood health status, adult, cardiovascular disease, China

## Abstract

Cardiovascular diseases (CVDs) are among the top health problems of the Chinese population. Although mounting evidence suggests that early childhood health status has an enduring effect on late life chronic morbidity, no study so far has analyzed the issue in China. Using nationally representative data from the 2013 China Health and Retirement Longitudinal Study (CHARLS), a Probit model and Two-Stage Residual Inclusion estimation estimator were applied to analyze the relationship between childhood health status and adulthood cardiovascular disease in rural China. Good childhood health was associated with reduced risk of adult CVDs. Given the long-term effects of childhood health on adulthood health later on, health policy and programs to improve the health status and well-being of Chinese populations over the entire life cycle, especially in persons’ early life, are expected to be effective and successful.

## 1. Introduction

Cardiovascular diseases (CVDs) are among the top health problems among the Chinese population. One in five adults are believed to suffer from CVDs, and about 3.5 million Chinese die of CVDs annually, accounting for 41% of deaths from any cause. The prevalence of CVDs is increasing steadily, suggesting an urgent need to develop adequate, preventive, and primary care in the Chinese countryside [1]. 

Mounting evidence suggests that early childhood life circumstances have an enduring effect on late life chronic morbidity [2,3,4,5,6,7,8]. Most of these studies are however from developed countries, such as the United States and the United Kingdom, and while a few studies have used data from developing countries to examine the long-term effects of childhood health on adult health outcomes, the evidence has been mixed. McEniry concluded that adverse endogenous (*in utero*) conditions, such as poor nutrition and infectious diseases were associated with heart disease and diabetes in elderly Puerto Rican adults [9]. Margolis investigated the relationship between five types of childhood morbidity and risk factors for cardiovascular disease among Guatemalan adults who experienced high levels of morbidity in childhood. Most types of childhood morbidity are associated with poorer health in early adulthood, independent of family background, adult socioeconomic status, and health behaviors. However, diarrheal diseases in later childhood were associated with lower levels of some risk factors, as measured by triglycerides and plasma glucose [10]. 

More empirical evidence is needed to figure out the associations between childhood health status and adult health outcomes, especially in developing countries [9]. China has its own unique features in terms of disease profiles. Historically, childhood health status in China was negatively affected by poor nutrition in the 1950s and 1960s. Beginning in 1959, China experienced a severe famine affecting the whole country. Over 30 million people died from starvation or severe malnutrition, and about 33 million births were either lost or postponed during the three-year period from 1959 to 1961, making this the largest famine in human history [11]. However, in the past 30 years, China has distinguished itself by rapid and sustained economic development. According to Chinese Statistical Yearbook 2015, from 1949 to 2014, life expectancy in China increased from 35 to 76.34 years. 

Several studies have found negative effects of malnutrition because of exposure to the great famine of 1959–1962 in early infancy on adult health in China [12,13,14]. To our knowledge, however, there is no study directly quantifying the long-term effects of childhood health on adult cardiovascular diseases in China. This study aimed to fill some of the literature/knowledge gap by analyzing the effect of childhood health status on the adult cardiovascular disease of people aged 45 to 64 in 2013. Especially, the study estimated the effects of health at the adolescence on adult cardiovascular diseases, which has not been done in previous research. If childhood health affects adulthood health in China, public health initiatives targeting childhood health will lead to long-term benefits.

## 2. Conceptual Framework

The pathways from childhood health status to adult health outcomes may be direct or indirect [15,16,17]. The so-called latency model asserts that childhood health is directly related to adult health outcomes through physical condition [15,16,18]. Negative events occurring during developmentally salient periods may permanently alter the trajectory of health over the life course. For example, negative events such as malnutrition may cause the pancreatic b cells to become (more) resistant to insulin [19,20,21]. Note that the development of pancreatic islets is known to last until the end of the adolescence [22]. Barker [19] also mentions the possibility that, in periods of severe under nutrition, the body sacrifices the growth of muscles and adipose tissues to focus on brain development, and that, in these conditions, muscles and adipose tissues have a reduced rate of glycolysis and altered insulin receptors, and that all this could be related to insulin resistance. 

The critical-period programming model emphasizes the importance of conditions in-utero, at birth, and in early infancy [23,24]. Health insults occurring at very early ages (even *in utero*) lead to disease pathologies later in life, lowering health trajectories over the life cycle [25]. People who suffer from poor nutrition *in utero*, at birth or in early infancy may develop “thrifty genes”, which adjust the body’s physiology and metabolism in order to promote survival in times of malnutrition [23]. Such thriftiness, however, may be harmful to health if poor nutrition is followed by good nutrition later in life, leading to higher risks of obesity, type 2 diabetes, and coronary heart diseases [19]. With rapid economic growth in developing countries comes a drastic shift in diets and lifestyle patterns. Adults who are now experiencing higher dietary energy intakes and more sedentary lifestyles were born when rates of poor maternal nutrition and low birth weight were high [26]. As a result, such individuals may be more likely to suffer from obesity, type 2 diabetes, and coronary heart disease. There is growing biological evidence that health conditions during such a critical period can change an individual’s body structure, physiology, and metabolism, permanently affecting adult health outcomes [27,28,29]. Thus the critical-period programming model predicts a positive relationship between childhood health and adult health. 

Other models emphasize indirect effects between child and adult health. The pathway model asserts that childhood conditions indirectly affect adult health through impaired adult socio-economic status [3,10,17]. Poor childhood health disrupts cognitive, affective, and social competencies [30], so children in poor health attain less education and have lower socioeconomic status as adults [7,17].

It has been well established that contemporaneous socioeconomic status is a powerful predictor of overall health status [31]. Low adult socioeconomic status reduces access to economic and social resources that promote health, leading to negative health outcomes [32]. Moreover, individuals engaged in healthy behaviors are more likely to be among the higher social classes and behaviors such as adequate sleep and exercise are robust predictors of better health outcomes [33,34]. Based on the well-established connections among socioeconomic status, health behaviors and health status, poor childhood health can indirectly lead to adverse adult health outcomes. The latency model, the critical-period programming model and the pathway model each predict a positive relationship between childhood health status and adult health outcomes, although the mechanisms through which childhood health affects adult health differ. In addition to understanding the direction of the relationship between childhood health and adult health, it is critically important from a policy perspective to gain insight into the magnitude of this relationship. That is an empirical issue, and a focus of this study.

## 3. Materials and Methods

### 3.1. Data

This study used data from the 2013 China Health and Retirement Longitudinal Study (CHARLS). The CHARLS is a nationally representative sample of Chinese residents aged 45 and above, which is publicly available [35]. CHARLS studied approximately 10,000 households in 150 counties/districts (a total of 450 villages/resident communities). The CHARLS adopts a multi-stage stratified Probability-Proportionate-to-Size (PPS) Sampling. The questionnaire covers seven sections: Demographic Background, Health Status and Functioning, Health Care and Insurance, Work, Retirement and Pension, Income, Expenditure and Assets, Interviewer Observation. The Institutional Review Board of Peking University provided ethical consent for the collection of CHARLS data.

Since the World War II and the 1946–1949 civil war in China undoubtedly affected child health significantly, and data related to death rates could not be collected for those periods, this study only included respondents who were born in or after 1949. Social economic status disease profiles differ greatly between people residing in urban and rural areas, and the severity of great famine varied widely between urban and rural areas, so this study focused on respondents who spent their whole life in rural areas. After we excluded the observations with missing values, 5735 observations were available for statistical analysis.

### 3.2. Measures

#### 3.2.1. Dependent Variables—Adult Cardiovascular Diseases

A category variable of adulthood doctor diagnoses of cardiovascular diseases was created based on the question: “Have you ever been diagnosed with heart attack, coronary heart disease, angina, congestive heart failure, or other heart problems by a doctor?” The variable equaled 1 if the respondent reported their childhood health as “YES”, and 0 otherwise. 

#### 3.2.2. Independent Variables

To measure childhood health, the CHARLS survey asked each respondent the following question: “How would you evaluate your health during childhood, up to and including age 15—Excellent, very good, good, fair, or poor?” We constructed a binary variable of “good childhood health” that equaled 1 if the response reports their childhood health as “excellent”, “very good”, or “good”, and 0 otherwise. 

Education and income are important and easily measured indicators of socioeconomic status. Education and log of household yearly income per capita were controlled. Adult educational attainment in the data was defined at three levels: primary school or below, junior high school, senior high school and college or above. We constructed three dummy variables for educational attainment, with primary school or below serving as the reference group. 

Two variables indicating health behavior were controlled. The first, smoking status, was classified as a current smoker or a non-smoker. The second, respondents were asked if they drank beer or any other alcoholic beverage during the previous 12 months. Those who had drunk in the past 12 months were identified as “current drinker” and were asked further questions about: frequency of drinking. Drink more than 2–3 times a week was considered as “drink frequently”.

Other CVDs risk factors such as hypertension and dyslipidemia were included. Respondents were asked if they had been diagnosed with Hypertension/Dyslipidemia (elevation of low density lipoprotein, triglycerides (TGs), and total cholesterol, or a low high density lipoprotein level) by a doctor. If the answer was “yes”, the variable to measure Hypertension/Dyslipidemia was assigned a value of 1, and otherwise 0. 

Demographic variables included gender (reference group: female), marital status (reference group: married with spouse present (common-law marriage was considered as married)) and age. Age quadric was also included to measure the non-linear effects of age on adult health outcome.

### 3.3. Analytical Techniques

Since having adulthood self-reported cardiovascular diseases was a categorical outcome, a Probit model was applied to analyze the relationship between childhood health status and adult health outcomes, controlling for respondents’ current socio-economic status, respondents’ current health behaviors and demographic variables [36]:

*H_adult_* = β_0_ + β_1_*H_child_* + β_2_*X* + ε (1)

where:

*H_adult_* = self-reported cardiovascular diseases;
*H_child_* = childhood health status before 15 years old;
*X* = a vector of other control variables;
ε = a disturbance term; and
β_0_β_1_β_2_ = coefficients to be estimated.

Self-reported childhood health status is potentially endogenous due to measurement error, unobserved omitted variables and/or simultaneity. Measurement error issues exist for both “self-reported” health status (the adult status as the dependent variable and the child health status as the major independent variable). Measurement error is one of the sources of endogeneity in the child health status measure. Childhood health status was unable to be directly measured when the respondent was younger (less than 15 years old), and must rely upon the respondent’s recollection about his or her childhood health. The respondent’s current health, social, and/or demographic factors may affect recalled responses about childhood health status, and some of these factors are unavailable in our database, leading to omitted variables problems. In addition, adult current health outcomes may also affect self-reported childhood health status, leading to a simultaneity issue.

One of main approaches to tackle the problem of endogeneity between childhood health and adult CVDs is to seek a valid instrument variable [37]. The Great Famine provides a unique historical context to estimate the role of an adverse experience played in the relationship between childhood health and adult health outcomes for two main reasons. First, the Great Famine was almost completely unexpected; China’s agricultural output kept growing at 3%–4% per year from 1952 to 1958, and the growth rate in 1958 was 2.8%. That growth reverted to a 14% contraction in 1959 and a further 13% decline in 1960 [38]. Second, China’s hukou (household registration) system constrained population mobility across counties, making it difficult for people to escape the Great Famine [12]. The instrument used in this study was the intensity of exposure to famine within subjects’ childhood period in the subject’s living area. That is, the instrument was the excess death rate for each observation being weighted according to the number of month exposure to famine. It was calculated as the difference between the annual provincial death rate from which was subtracted from the mean provincial death rate between 1959 and 1962 following Chen and Zhou [12]. Respondents bore in different months spent different amounts of time in their childhood period. For example, an individual born in October 1960 had 3 months in 1960, 12 months in 1961, 12 months in 1962, there are 180 months in his/her childhood up to 15 years of age, so the instrument will equal to 3/180 × “the excess death rate in 1960 in the subject’s living area (%)” + 12/180 × “the excess death rate in 1961 in the subject’s living area (%)” + 12/180 × “the excess death rate in 1962 in the subject’s living area (%)”. The instrumental variable ranged from −1.65 to 1.39 in our study; the mean value was 0.20. This instrumental variable should be correlated with childhood health status, but should not directly affect adult cardiovascular diseases and only work through its effects on childhood health indirectly. As expected, the instrumental variable in the living area was negatively correlated with the childhood health status (*p* < 0.01). Further, the instrumental variable had no direct influence on adult health outcomes. The famine studies have shown a negative effect of poor nutrition on childhood health and long-term consequences for adult health [12]. A number of statistical tests of our instruments also indicate that our instrument is valid. Even if we were not able to implement the two-stage least squares estimation due to the binary nature of adult health outcomes in the second stage, the test of the joint significance of the instruments in the two-stage least squares estimation yields an *F*-statistic of 45.41 (*p* < 0.01). A number of statistical tests of our instruments also indicated that our instrument is valid in terms of the excluded-instrument test, under-identification test, weak-identification test and weak-instrument-robustness test. These results are available from the authors on request.

Since an adult cardiovascular disease was categorical, a Two-Stage Residual Inclusion (2SRI) Estimation was applied. The 2SRI estimation extends the two-stage least squares (2SLS) linear modeling framework with the dependent variable in the second stage to be nonlinear outcomes. 2SRI estimation has been widely used to address endogeneity in health economics and health services research. It has been shown that 2SRI yields results that are consistent and efficient [39]. The first stage equation is estimated as follows:

*H_child_* = α_0_ + α_1_*IV* + α_2_*X* + *u* (2)

where:

*IV* = instrumental variables;
*u* = residual;
α_0_α_1_α_2_ = coefficients to be estimated.

The second stage equation can be obtained by adding the residual from the first stage into Equation (1) as follows:

(3) $$H_{adult}=\beta_{0}+\beta_{1}H_{child}+\beta_{2}X+\beta_{3}\overset{\wedge }{\mu }+\varepsilon$$

Since the childhood health status variable in the first stage was binary, we had employed Probit estimation and the generalized residuals from the Probit estimation in the first stage were inserted into the second stage [40]. Because the nonlinear system is estimated in two steps, the standard errors produced are incorrect, as they fail to account for the stochastic nature of the estimated residual terms. Therefore, we used bootstrapping with 1000 iterations to calculate correct standard errors [41]. The statistical software used in this paper was Stata 14.

## 4. Results

Table 1 shows the descriptive summary of variables by sex. In total 4.17% of respondents reported they had been diagnosed with cardiovascular diseases; as for childhood health status, a total of 72.5% of respondents reported that their childhood health up to the age of 15 was excellent, very good, or good. Compared to women, men were slightly more likely to report good child health (men: 72.9% *vs.* women: 72.02%) and diagnosed with cardiovascular diseases (men: 4.5% *vs.* women: 3.8%).

**Figure 1:**
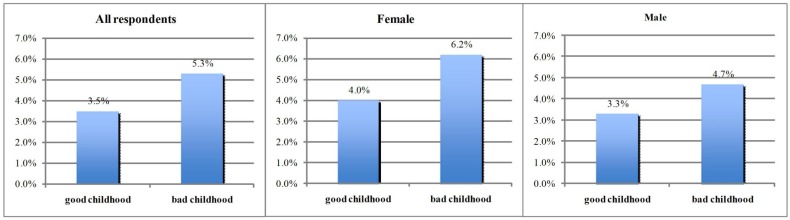
The morbidity of CVDs by childhood health.

Figure 1 shows a negative association between good childhood health status and adult cardiovascular diseases. We find that both men and women respondents with poor childhood health were more likely to report bad cardiovascular diseases (*p* < 0.01). 

Table 2 shows the multivariate regression results for the effects of childhood health on adult cardiovascular diseases, controlling for socio-economic status, health behaviors as well as demographic characters. 

Good childhood health status was negatively associated with CVDs. Respondents with good childhood health, versus those with bad childhood health, were associated with reduced risk of adult CVDs by 0.24 (*p* < 0.01) without correcting for endogeneity issue. The results also showed the multivariate regression results for the effects of childhood health on adult cardiovascular diseases by gender. When analyses were stratified by sex, the results were consistent. Compared with individuals having bad childhood health, individuals having good childhood health were related to reduced risk of adult CVDs by 0.27 for male (*p* < 0.01), 0.2 for female (*p* < 0.05). After correcting for endogeneity issues, good childhood health status was found to be negatively associated with CVDs, and the effects were stronger. Respondents with good childhood health were less likely (by 2.1-fold) to have CVDs than their counterparts with bad childhood health.

## 5. Discussion

Using the 2013 CHARLS, this study examined the long-term effects of childhood health status on adult CVDs in rural China. Our results are consistent with the main previous research [2,3,4,5]. A strong association of good childhood health with reduced risk of adult CVDs in China was observed, even after controlling for demographic, family background, and adult characteristics. The effects of childhood health on adult health are similar to the results after correcting for endogeneity. In China, the latency model and the pathway models support the observed relationship between childhood health status and adult health outcomes. Childhood health measures are related to adult health outcomes, independent of adult socioeconomic status and health behaviors. 

Our results have several health policy implications for China. First, an integrated health policy on cardiovascular diseases prevention and control should be based on the premise of maximizing health and well-being over the entire life cycle rather than only focusing on the health of specific age groups. The fact that childhood health is essential to achieve the goal of cardiovascular disease prevention and control suggests that policy interventions for cardiovascular disease should work throughout the entire life cycle, starting in childhood. Second, adult health inequalities may in part due to childhood health status. As a result, policies aimed at achieving health equality among the adult population should recognize the importance of achieving health equality during childhood years. Policy makers should be forward-looking in investing in childhood health. Investing in children’s health seems quite rational for both individuals and societies: individuals could obtain longer and healthier lives while the collective costs of health care to future generations of elderly would be controlled. This is especially important given that the future cohorts of children are expected to live longer than the present generation.

This paper is not without limitations. Our work on childhood health in CHARLS relied on a retrospective self-evaluation using a standard five-point scale (excellent, very good, good, fair, or poor) of the general state of health when one was up to age 15 proxies for health during the childhood years. Self-reported childhood health status is potentially endogenous due to measure error or/and simultaneity [38]. The effects of childhood health on adult health were stronger when endogeneity issue was corrected, which may be partly explained by the possibility that individuals with poor adulthood health were more likely to report poor childhood health conditions. However, self-report health is a widely-used variable to measure health outcome and has been validated as providing a good summary measure of overall childhood health including respiratory diseases, heart disease, childhood diabetes, headaches and migraines, ear infections, and stomach problems [42]. Smith also used self-report health to measure childhood health status that modeled the consequences of childhood health on adult health and socio-economic status outcomes with CHARLS pilot data and validated this variable in the context of China [43]. 

## 6. Conclusions

This is the first study to quantify long-term relationships between childhood and adult cardiovascular disease using nationally-representative data from China. Good childhood health is associated with reduced risk of adult CVDs. An integrated health policy based on the premise of maximizing health and well-being over the entire life cycle should be highly recommended. Policy makers should be forward-looking in investing in childhood health. 

## Figures and Tables

**Table 1:** Descriptive statistics.

Factor	All Respondents (*n* = 5735)	Male (*n* = 2764)	Female (*n* = 2971)
Morbidity of cardiovascular diseases	4.17%	4.5%	3.8%
Good childhood health	72.5%	72.9%	72.02%
Education level			
Primary school and below	69.1%	67.6%	70.7%
Junior high school	21.6%	27.5%	15.3%
High school and above	9.3%	12.5%	5.9%
Household income per capita (1000 Yuan) ^#^	8.49 (18.86)	8.91 (18.92)	8.16 (18.45)
Hypertension	19.9%	18.5%	21.4%
Dyslipidemia	7.29%	6.8%	7.84%
Age	54.91	55.03	54.66
Gender	48.2%		
Marital status	92.7%	93.6%	91.7%
Smoker	30.06%	53.3%	5.1%
Frequent drinker	26.99%	45.9%	6.7%

^#^ Mean, SD.

**Table 2:** The effects of childhood health status on adult cardiovascular diseases.

Factor	All Respondents		Male		Female	
	2SRI Coef. (Std.)	Probit Coef. (Std.)	2SRI Coef. (Std.)	Probit Coef. (Std.)	2SRI Coef. (Std.)	Probit Coef. (Std.)
Good childhood health	−2.10 ***	−0.24 ***	−2.59 *	−0.27 ***	−1.73 **	−0.20 **
	(0.74)	(0.06)	(1.38)	(0.09)	(0.06)	(0.09)
Residual	1.13 **	-	1.38 *	-	0.99 *	-
	(0.44)	-	(0.82)	-	(0.51)	-
Household income	−0.01	−0.01	−0.01	−0.01	−0.01	−0.01
	(0.01)	(0.01)	(0.01)	(0.01)	(0.01)	(0.01)
Dyslipidemia	0.21 *	0.31 ***	0.22	0.42 ***	0.09	0.17
	(0.11)	(0.11)	(0.16)	(0.15)	(0.24)	(0.18)
Hypertension	0.28	0.1 *	0.27	0.01	0.28 *	0.22 *
	(0.22)	(0.08)	(0.29)	(0.26)	(0.15)	(0.11)
Age	−0.001	0.20	−0.002	−9.67 × 10^−5^	0.15	0.46 *
	(0.002)	(0.19)	(0.002)	(0.002)	(0.37)	(0.28)
Age quadric	−0.001	−0.002	−0.001	−0.001	−0.003	−0.004
	(0.001)	(0.001)	(0.002)	(0.002)	(0.003)	(0.003)
Gender	−0.0297	0.150 *	-	-	-	-
	(0.0945)	(0.0906)	-	-	-	-
Junior high school	0.01	−0.03	0.02	0.29	−0.03	−0.05
	(0.09)	(0.08)	(0.18)	(0.18)	(0.13)	(0.10)
High school	0.12	0.03	0.2	0.50 ***	0.09	−0.10
	(0.14)	(0.11)	(0.21)	(0.16)	(0.15)	(0.13)
Marital status	0.08	0.02	0.08	0.08	0.09	0.01
	(0.10)	(0.13)	(0.17)	(0.16)	(0.23)	(0.19)
Smoker	−0.17 *	−0.002	0.25	0.29	−0.18	−0.12
	(0.09)	(0.08)	(0.19)	(0.18)	(0.11)	(0.08)
Frequent drinker	0.14	0.45	−0.01	−0.07	0.85 **	0.46
	(0.11)	(0.34)	(0.23)	(0.18)	(0.42)	(0.36)

Coef. (Std.): Coefficient and standard errors are shown; * *p* < 0.1; ** *p* < 0.05; *** *p* < 0.01.
